# Exceptional Packing Density of Ammonia in a Dual-Functionalized Metal–Organic Framework

**DOI:** 10.1021/jacs.1c01749

**Published:** 2021-04-22

**Authors:** Christopher Marsh, Xue Han, Jiangnan Li, Zhenzhong Lu, Stephen P. Argent, Ivan da Silva, Yongqiang Cheng, Luke L. Daemen, Anibal J. Ramirez-Cuesta, Stephen P. Thompson, Alexander J. Blake, Sihai Yang, Martin Schröder

**Affiliations:** † Department of Chemistry, University of Manchester, Manchester, M13 9PL, U.K.; ‡ School of Chemistry, University of Nottingham, Nottingham, NG7 2RD, U.K.; § ISIS Neutron and Muon Source, Rutherford Appleton Laboratory, Oxford, OX11 0QX, U.K.; ∥ Neutron Scattering Division, Neutron Sciences Directorate, Oak Ridge National Laboratory, Oak Ridge, Tennessee 37831, United States; ⊥ Diamond Light Source, Harwell Science Campus, Oxfordshire, OX11 0DE, U.K.

## Abstract

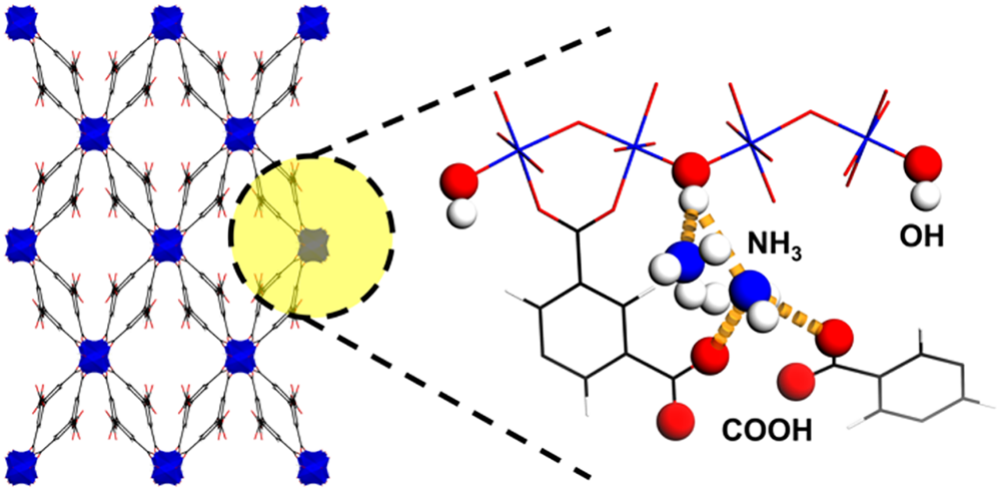

We report the reversible adsorption of ammonia (NH_3_) up to 9.9 mmol g^–1^ in a robust Al-based metal–organic framework, MFM-303(Al), which is functionalized with free carboxylic acid and hydroxyl groups. The unique pore environment decorated with these acidic sites results in an exceptional packing density of NH_3_ at 293 K (0.801 g cm^–3^) comparable to that of solid NH_3_ at 193 K (0.817 g cm^–3^). *In situ* synchrotron X-ray diffraction and inelastic neutron scattering reveal the critical role of free −COOH and −OH groups in immobilizing NH_3_ molecules. Breakthrough experiments confirm the excellent performance of MFM-303(Al) for the capture of NH_3_ at low concentrations under both dry and wet conditions.

## Introduction

Over 150 million tonnes of NH_3_ are produced each year primarily as fertilizer, consuming 2% of the world’s energy.^1^ Agricultural activities have led to significant emissions of NH_3_, making it one of the five most damaging air pollutants, along with particulate matters, NO_*x*_, SO_2_, and non-methane volatile organic compounds.^2^ NH_3_ can also combine with urban NO_*x*_, thus contributing to the formation of smog.^3^ Nonetheless, as one of the most highly produced inorganic chemicals in the world, NH_3_ is also a candidate for the storage and distribution of H_2_ for the implementation of the hydrogen economy.^4^ Possessing very high gravimetric (∼17 wt % or 5.8 kWh/kg) and volumetric (∼0.105 kg/L or 3.6 kWh/L at 25 °C) densities of H_2_ energy, NH_3_ can be produced cheaply and transformed directly to yield H_2_ with N_2_ as the byproduct.^5^ However, several prerequisites need to be fulfilled for any practical use, including safe and efficient storage media with high volumetric uptakes, a facile cracking process, and an effective capture and removal system since even 1 ppm of NH_3_ can poison proton exchange membrane fuel cells.^6^ A variety of materials have been investigated as storage and capture media for NH_3_, such as activated carbons,^7^ zeolites,^8,9^ mesoporous silica,^10,11^ and organic polymers.^12,13^ Although porous materials as regenerable NH_3_ capture systems have been demonstrated, many are limited by factors such as low storage capacity, irreversible uptake, and/or low packing density of stored NH_3_, which restrict their on-board applications due to the additional volume required for the storage system.

Metal–organic framework (MOF) materials possess high porosity and surface area, and have been investigated extensively for the adsorption and storage of small molecule gases.^14^ However, the highly reactive nature of NH_3_ makes its storage challenging,^15−17^ with few MOFs demonstrating the required high stability over multiple adsorption–desorption cycles.^18−20^ Previous studies have examined the role of functionality and the role of acidic sites in improving adsorption of NH_3_ in porous organic polymers.^21−23^ This role has also been confirmed by computational studies.^24^ Adsorption of NH_3_ in MFM-300(Al), a hydroxyl-decorated MOF, at ambient conditions shows a packing density in the pore of 0.62 g cm^–3^ approaching that of liquid NH_3_ (0.681 g cm^–3^) at 240 K.^18^ Here, we report the highly efficient storage of NH_3_ in dual-functionalized MFM-303(Al) incorporating pores decorated with both free carboxylic acid and hydroxyl groups. These act as binding sites for NH_3_ to yield a record-high packing density of NH_3_ (up to 0.88 g cm^–3^) compared to all porous materials known to date. The adsorption domains and binding dynamics of NH_3_ in MFM-303(Al) have been investigated by *in situ* synchrotron X-ray diffraction and inelastic neutron scattering (INS) coupled with computational modeling.

## Materials and Methods

### Chemicals

All materials were purchased from commercial suppliers and used without further purification.

### Synthesis of MFM-303(Al), [Al(OH)(C_16_O_8_H_8_)](H_2_O)_2_

**Scheme 1:**
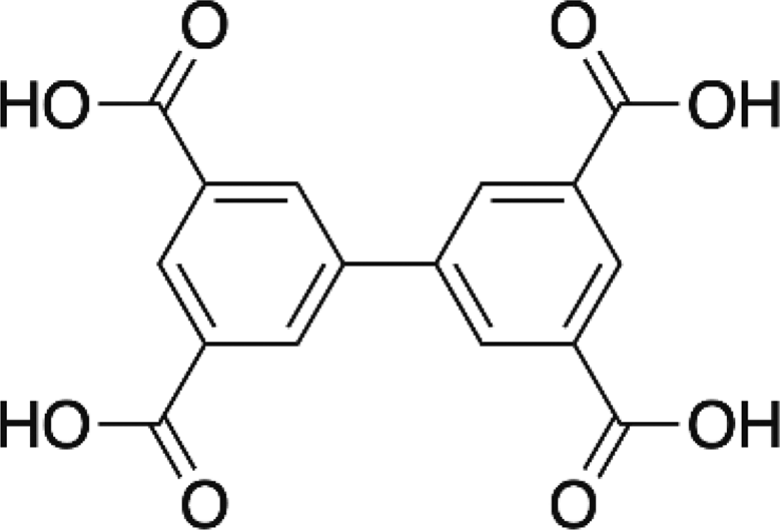
Chemical Structure of Biphenyl-3,3′,5,5′-Tetracarboxylic Acid

Biphenyl-3,3′,5,5′-tetracarboxylic acid (60 mg, 0.182 mmol, Scheme 1) and AlCl_3_ (121.2 mg, 0.909 mmol) were combined with water (10 mL) acidified with 2% HCl (2 mL) in a PTFE-lined stainless steel autoclave. The autoclave was sealed and heated for 3 days at 210 °C. The white crystalline product was isolated by filtration, washed with water, and then dried in air. Yield: 59.1 mg (79.6% based on ligand). Elemental analysis [Al(OH)(C_16_O_8_H_8_)](H_2_O)_2_ (% calc/found): C 46.65/46.90, H 3.29/3.32, N 0.0/0.0. Selected IR (ATR): *v*/cm^–1^: 3085(w), 1683(m), 1615(m), 1579(s), 1409(m), 1246(m), 1167(m), 1089 (m), 986(s), 803(m), 764(s), 648(m).

### X-ray Crystallographic Study of MFM-303(Al)

Single crystal X-ray diffraction data of MFM-303(Al) were collected at 120 K using synchrotron radiation at Beamline I19 of Diamond Light Source, equipped with three-circle goniometer and Rigaku Saturn 724+ CCD detector (λ = 0.68890 Å, double crystal monochromator with Si 111 cryo-cooled crystals). Details of the data collection are included as part of the Crystallographic Information File (CIF) in the Supporting Information, with crystallographic data and details of the structure refinements summarized in Supplementary Table S1.

### Gas Adsorption Isotherms and Breakthrough Experiments

Gravimetric isotherms for CO_2_, N_2_ and NH_3_ were measured on an Xemis system (Hiden Isochema) under an ultrahigh vacuum in a clean system with a diaphragm and turbo pumping system. All gases were of ultrapure research grade (99.9999%) and purchased from BOC. The acetone-exchanged MOF (∼70 mg) was outgassed at 393 K over 18 h prior to measurement.

Breakthrough experiments were undertaken using a fixed-bed tube (7 mm diameter, 120 mm length) packed with 420 mg of MFM-303(Al). The sample was activated by heating under a flow of He for 1 day at 423 K. The fixed bed was cooled to 298 K using a water bath, and a breakthrough experiment was performed with a flow of NH_3_ (833 ppm) diluted in He at atmospheric pressure with a flow rate of 48 mL min^–1^. For the test under humid conditions, the fixed bed was pre-saturated with water using wet He with a flow rate of 40 mL min^–1^ until breakthrough of water was observed, at which point the flow of NH_3_ diluted in He was turned on, giving a combined flow rate of 48 mL min^–1^. The concentration of NH_3_ was determined by mass spectrometry and compared with the inlet concentration C_0_, where C/C_0_ = 1 indicates complete breakthrough.

### High Resolution Synchrotron Powder X-ray Diffraction and Structure Determination of Binding Domains for Adsorbed NH_3_ Molecules.

High resolution *in situ* powder synchrotron X-ray diffraction (PXRD) data were collected at Beamline I11 of Diamond Light Source using multi-analyzing crystal (MAC) detectors and monochromated radiation (λ = 0.825774 Å) and an *in situ* gas cell system. The powder sample was loaded into a capillary tube of 0.7 mm diameter, degassed at 393 K, and loaded with NH_3_ at 298 K, and the data collection was carried out at 273 K. The structure solution was initially determined by considering the structure of the bare MFM-303(Al) framework, and the residual electron density maps were further developed from subsequent difference Fourier analysis using TOPAS. The final structural refinement of MFM-303(Al)·4.36NH_3_ was undertaken using the Rietveld method with isotropic displacement parameters for all atoms. Upon desolvation and gas loading, changes in the intensities of Bragg peaks were observed indicating the adsorption of NH_3_ into the material. Upon desolvation, a slight phase change was observed in MFM-303(Al); despite our best efforts, we were unable to determine the structural basis of this change. Upon gas loading, the original phase returned.

### Inelastic Neutron Scattering

Inelastic neutron scattering (INS) measurements were obtained using the VISION spectrometer at the Spallation Neutron Source, Oak Ridge National Laboratory, Oak Ridge, Tennessee, USA. The sample was loaded in an aluminum can and degassed at 393 K over 24 h, and data were collected at 5 K. NH_3_ was dosed in at room temperature and the sample cooled to 5 K before data collection.

## Results and Discussion

### Synthesis and Crystal Structure Analysis

**Figure 1:**
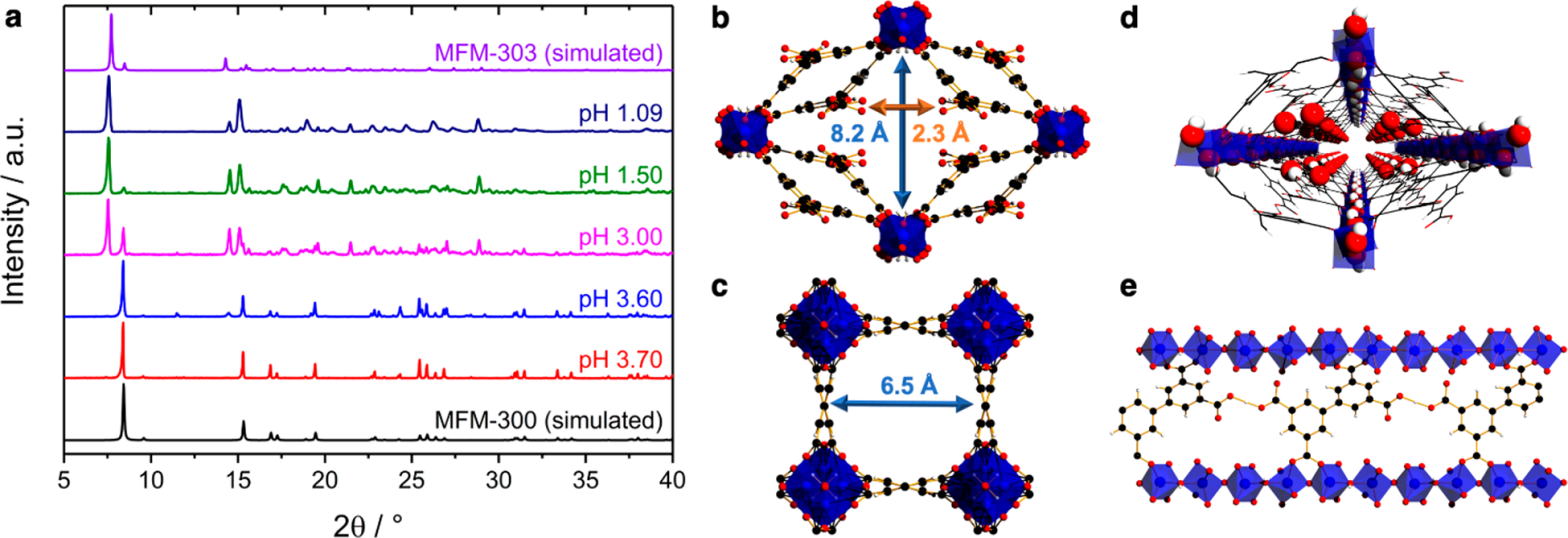
(a) PXRD patterns of products formed at different pH in the synthesis of MFM-303(Al) and MFM-300(Al). Pore dimensions for (b) MFM-303(Al) and (c) MFM-300(Al) viewed along the crystallographic *c* axis. (d) Perspective view of MFM-303(Al) with the oxygen atoms of free carboxylic acid groups and μ–OH highlighted. (e) View of MFM-303(Al) showing hydrogen bonding. Aluminium, blue; carbon, black; oxygen, red; hydrogen, white; [AlO_4_(OH)_2_], blue octahedra).

Constructed from the same ligand and metal salt as MFM-300(Al), MFM-303(Al) was obtained under more acidic conditions (pH < 3.0), which partially hinders the coordination of carboxylic acid groups to Al(III) and results in the presence of free −COOH groups in the product (Figure 1). MFM-303(Al) can be synthesized in pure phase form by carrying out the synthesis at pH of 1.09 and is isolated as rod-shaped colorless single crystals. Single crystal X-ray diffraction confirms that MFM-303(Al) crystallizes in the monoclinic space group *C*2/*c*, with each Al(III) center coordinated octahedrally to four oxygen atoms from carboxylate groups and two from bridging hydroxyl groups in a mutually *trans* orientation. Only two carboxylate groups of each tetracarboxylic ligand are bound to Al(III) centers, while the other two remain uncoordinated and form intramolecular hydrogen bonds [O···O= 2.637(14) Å] with neighboring ligands. This suggests a moderate-to-strong hydrogen bonding interaction with the hydrogen atom bridging two oxygen atoms.

The one-dimensional [AlO_4_(OH)_2_]_∞_ chains running along the *c* axis are bridged by partially deprotonated linkers along the *a* and *b* axes affording a 3D open framework structure. The formation of a unique “double-glazed” pore wall causes distortion of the pore geometry from 6.5 × 6.5 Å^2^ in MFM-300 (Al) to 8.2 × 2.3 Å^2^ in MFM-303(Al) accompanied by a 56% reduction of the pore volume from 0.433 to 0.191 cm^3^ g^–1^, as determined using PLATON^25^ with a default probe radius of 1.2 Å. Interestingly, this distortion creates additional domains functionalized with acidic sites.

### NH_3_ Sorption Analysis

**Figure 2:**
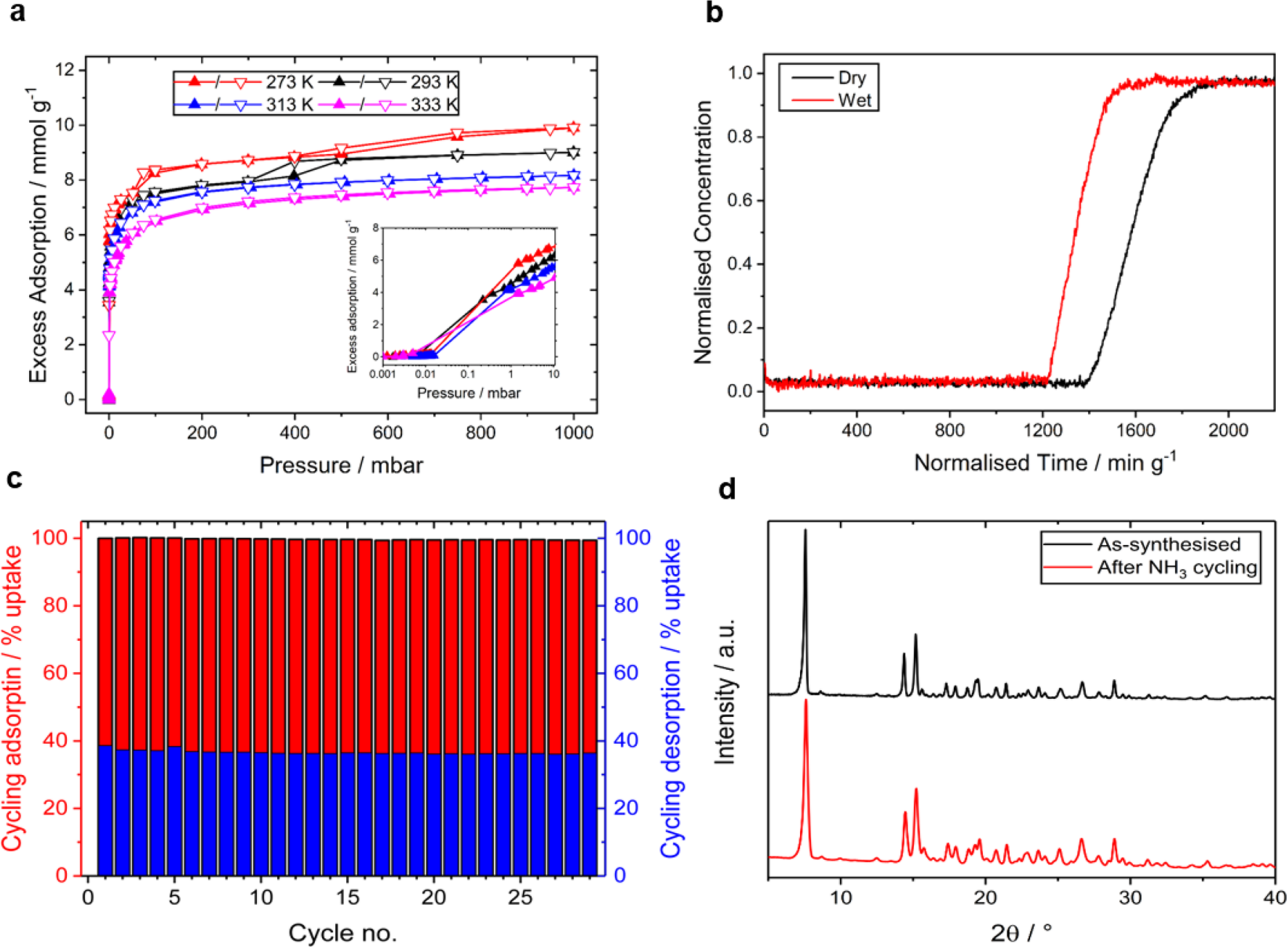
(a) Adsorption isotherms for NH_3_ in MFM-303(Al) at 273–333 K with inset highlighting the low-pressure region. (b) Breakthrough curves for NH_3_ (833 ppm) diluted in He under dry and wet (83% relative humidity) conditions at 298 K and 1 bar. (c) Cyclic adsorption–desorption of NH_3_ in MFM-303(Al). (d) PXRD patterns of fresh MFM-303(Al) and sample after cycling experiments with NH_3_ without heating treatment.

Adsorption isotherms for NH_3_ in MFM-303(Al) were recorded up to 1 bar between 273 and 333 K (Figure 2a). The isotherms exhibit ultrastrong adsorption at low pressure with an uptake of 4.5 mmol g^–1^ at 1 mbar, 293 K. After a plateau in the uptake, a stepped increase is observed, most notably for isotherms at 283, 293, and 298 K (Figure S7), before reaching another plateau. Apparent steps in isotherms have been observed previously in MIL-53(Al) assigned to framework transitions.^26,27^ In contrast, the steps in the isotherms for MFM-303(Al) are less pronounced, suggesting relatively minor structural distortion or rearrangement of adsorbed NH_3_ molecules within the pore (see below).

Although MFM-303(Al) has a lower NH_3_ uptake (9.9 mmol g^–1^ at 1 bar, 273 K, Figure 2a) than MFM-300(Al) (15.7 mmol g^–1^), the availability of free −COOH and μ–OH sites in the former results in a more efficient packing of NH_3_ molecules to give a record-high NH_3_ density of 0.881 g cm^–3^. The uptake at 293 K reveals only a minor reduction (9.1%) in uptake with a packing density of 0.801 g cm^–3^. This result is highly unusual considering the density of solid NH_3_ at 193 K is 0.817 g cm^–3^. Even using a probe radius of 1.0 Å in PLATON,^25^ a pore volume of 0.221 cm^3^ g^–1^ is obtained for MFM-303(Al) based upon the as-synthesized structure with free water removed from the pores. This gives packing densities of NH_3_ of 0.762 (273 K) and 0.693 g cm^–3^ (293 K), compared with a density for liquid NH_3_ of 0.681 g cm^–3^. For practical applications, the volume of the bulk sample and packing efficiency are important, and these factors are currently under further investigation. Due to the rapid uptake of NH_3_, it was not possible to calculate accurately the heat of adsorption (*Q*_*st*_*)* from the obtained isotherms. Instead, the *Q*_*st*_ was determined by differential scanning calorimetry (DSC) yielding a value of 61.5 kJ mol^–1^, which is notably higher than that (∼40 kJ mol^–1^) for MFM-300(Al) (Figure S11).

### Determination of the Binding Sites for Adsorbed NH_3_

**Figure 3:**
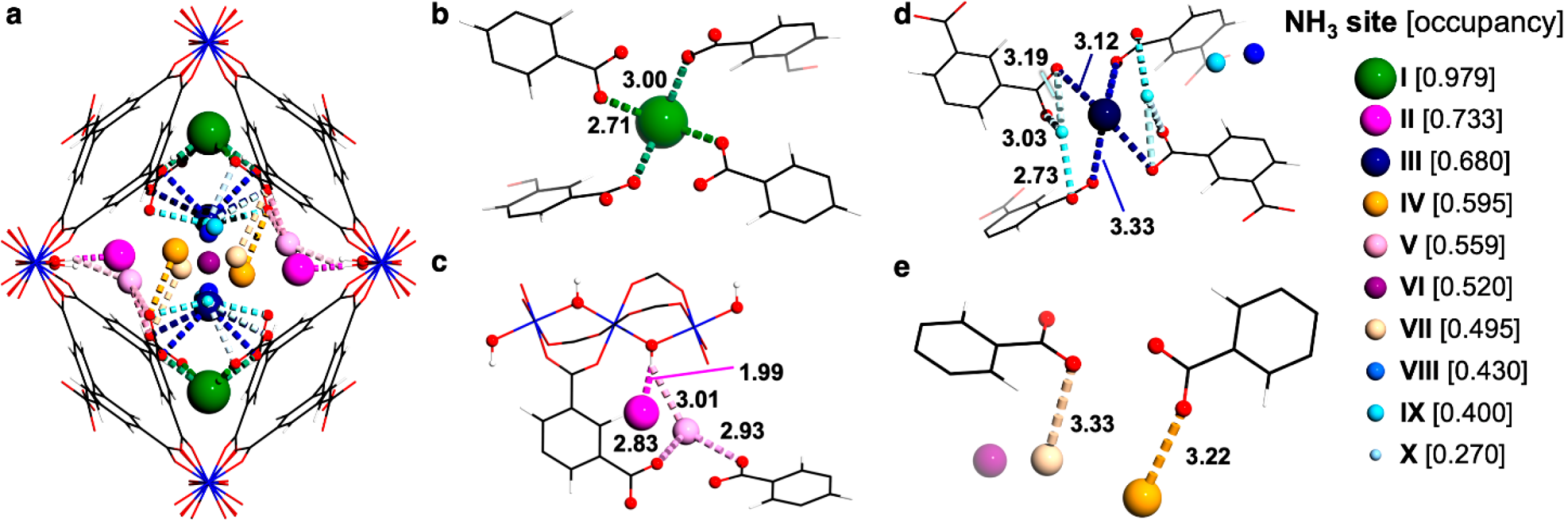
Location of binding domains of NH_3_ within MFM-303(Al) determined by high-resolution synchrotron PXRD. Framework shown in wireframe mode, and N atoms shown in ball and stick mode with the radius of the sphere proportional to the occupancy (shown in square brackets) at that position; aluminum, blue; carbon, black; oxygen, red; hydrogen, white; nitrogen, various colors dependent on binding site. (a) View of all locations of NH_3_ within the pore. (b) Binding site I showing the medium-strong interactions of NH_3_ with free −COOH groups of the framework. (c) Binding sites II and V showing interactions of NH_3_ with the μ–OH and −COOH of the framework, respectively. (d) Binding sites III, VIII, and IX showing interactions of NH_3_ with free −COOH groups of the framework. (e) Binding sites IV, VI, and VII showing interactions of NH_3_ with free −COOH groups of the framework. All distances shown are in Å.

Rietveld refinement of the synchrotron powder X-ray diffraction data for NH_3_-loaded MFM-303(Al) confirms the highly efficient packing of NH_3_ molecules within the pores. Unlike NH_3_@MFM-300(Al), where the main binding interaction originates from the bridging O–H groups at the four corners of its square-shaped channel, MFM-303(Al)·4.36NH_3_ exhibits a four-pointed star-shaped pore incorporating dual-functionality with unbound −COOH and μ–OH sites interacting with NH_3_ molecules (Figure 3). The primary binding site of NH_3_^I^ is located within the longer arms of the star and is anchored simultaneously by four −COOH groups, exhibiting a full occupancy with O···N distances of 2.71(1) Å. The presence of ultrastrong binding of NH_3_ has been confirmed by temperature-programmed desorption (TPD), where an uptake of ∼2.4 mmol g^–1^ was released at ∼150–200 °C (Figure S12). The shorter arms of the star are occupied by NH_3_^II^ (occupancy of 0.733), held in position through hydrogen bonding with μ–OH groups [O···N = 2.91(2) Å]. The center of the pore is filled in order with NH_3_^III–V^, which are stabilized through hydrogen bonding with −COOH groups [O···N = 2.83(2)−3.33(4) Å]. Interestingly, even the centermost space of the pore is occupied by NH_3_^VI^ (occupancy of 0.520), which does not interact directly with functional groups on the pore wall but is stabilized by the interaction with adjacent NH_3_^VIII^ molecules through hydrogen bonding [N^VI^···N^VIII^ = 2.36(3) Å]. The close intermolecular distances between NH_3_ molecules at all sites suggest significant hydrogen bonding with N···N distances ranging from ∼3 to 4 Å (Figure S15), comparable to that (3.378 Å) observed in the structure of solid NH_3_.^28^ Thus, the structural analysis of MFM-303(Al)·4.36NH_3_ has rationalized the exceptional packing density of NH_3_ in MFM-303(Al) as indicated by gas adsorption studies.

The structure of NH_3_-loaded MFM-303(Al) also gives insight into the stepped profile of the isotherms. Assuming the filling of the pore correlates to the crystallographic occupancies of NH_3_ sites, a loading of ∼7 mmol g^–1^ corresponds to sites I–V being occupied. These sites are located close to the −COOH and −OH groups in the pores, which are the favorable binding sites. After this point, site VI, located at the center of the pore, is filled, which likely gives rise to a stepped increase in uptake; it is not located close to any functional groups, and so there is not a strong preference for pore filling. Once this site is occupied, sites VII–X, which are located near other sites with higher occupancy, are filled, accounting for a slight increase in uptake as the pressure approaches 1 bar.

### Analysis of Host–Guest Binding Dynamics

**Figure 4:**
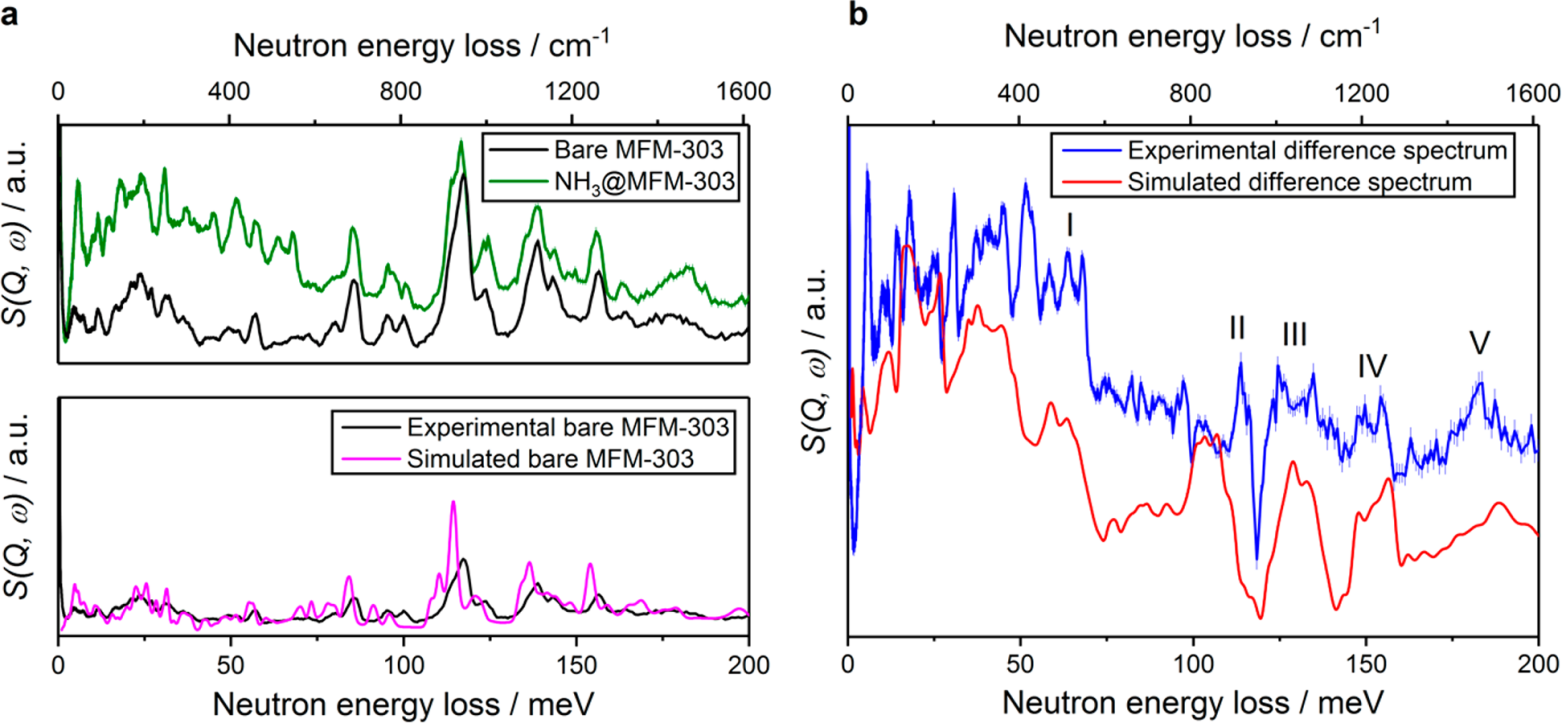
(a) Experimental INS spectra of bare and NH_3_-loaded MFM-303(Al) (top); experimental and simulated INS spectra of bare MFM-303(Al) (bottom). (b) Difference INS spectra.

*In situ* INS, coupled with DFT calculations, was employed to gain insight into the dynamics of NH_3_ loading in MFM-303(Al) at 1.0 NH_3_ per Al. The difference spectra were obtained by subtracting the background spectrum of the MOF from that of the NH_3_-loaded material. INS features for adsorbed NH_3_ were observed at the low-energy region (5–52 meV) and showed: (i) translational motions of adsorbed NH_3_ (5–12 meV) sited perpendicular to the N···H–O_bridging_ bond; (ii) librational motion of NH_3_ (14–31 meV) around its *C*_3_ axis; (iii) tilting modes of NH_3_ (35–52 meV). Peaks at higher energy (58–189 meV) reflect the changes to the vibrational motions of the H-centers of the framework upon adsorption of NH_3_. Good agreement between the experimental and calculated spectra allows the interpretation of five main features in the difference spectra as labeled I–V in Figure 4. Peaks in region I (58–68 meV) can be attributed to the deformational modes of the benzene ring, and the increased intensity observed upon binding of NH_3_ is due to the movement of adsorbed NH_3_ molecules toward the benzene ring, thus enhancing these modes. Peak II (113–116 meV) and the following dip are caused by a red shift (∼0.6 meV) of the main peak in Figure 4a at this energy. This peak has contributions from H–O rocking out of the Al–O–Al plane as well as out-of-plane wagging of the aromatic −CH groups in the ligand. The red shift occurs due to NH_3_ hindering the motion of the −OH and −CH groups. A similar explanation can be applied to peak III (124–134 meV) and the following dip, peak IV (147–154 meV) and the dip, and peak V (182–187 meV). In these cases, the red-shifted modes are the in-plane scissoring of H atoms at C6, C6’ positions of the ligand (III) and the in-plane wagging of H atoms at C2, C4 positions on the ligand (IV), and the in-plane scissoring of C–H at C2, C6 positions of the ligand (V) (Figure S13). These results are entirely consistent with the structural model of NH_3_-loaded MFM-303(Al) derived by PXRD.

### Dynamic Uptake of NH_3_

The dual-functionalized pore environment of MFM-303(Al) results in extremely high adsorption of NH_3_ at low pressure (Figure 2a). For example, an uptake of 6.0 mmol g^–1^ of NH_3_ can be achieved at 2.0 mbar in MFM-303(Al) at 273 K, corresponding to 60% of the saturated uptake at 1 bar. By contrast, MFM-300(Al) showed only 1.9 mmol g^–1^ uptake, 12% of that at 1 bar. This observation is particularly desirable in applications for NH_3_ capture, such as in personal protective equipment and elimination of air pollution. The ability of MFM-303(Al) to remove NH_3_ at low concentrations is confirmed by dynamic breakthrough experiments where 833 ppm of NH_3_ in He was passed through a fixed bed packed with MFM-303(Al) at 298 K under both dry and humid (83% relative humidity) conditions (Figure 2b). Strong retention of NH_3_ was observed in both cases, and the dynamic adsorption capacity of NH_3_ was calculated to be 2.9 and 2.4 mmol g^–1^ under dry and humid conditions, respectively.

The difference in capacity when wet NH_3_ is used is likely due to competitive water uptake inside the MOF. A water sorption isotherm (Figure S8) reveals that MFM-303(Al) has the capacity to adsorb a similar quantity of water to NH_3_. However, the isotherm does not exhibit the same rapid uptake with pressure and shows a slight inflection. The as-synthesized structure contains water molecules crystallographically located at three sites within the pores. These include positions at both the longer and shorter arms of the star, analogous to sites I and II observed for NH_3_ and with similar intermolecular distances to −COOH and −OH groups (Figure S16).

The static isothermal NH_3_ uptake of MFM-303(Al) under similar conditions (0.8 mbar and 298 K) is 4.2 mmol g^–1^ and implies that ∼70% of its thermodynamic capacity is achieved under dynamic conditions by virtue of fast adsorption kinetics. Complete regeneration of the NH_3_-loaded MFM-303(Al) can be achieved by heating at 353 K under dynamic vacuum, and full retention of the structure was confirmed after 29 cycles of NH_3_ adsorption–desorption (Figure 2c,d). The dynamic uptake capacity is comparable to other MOFs (Table S3), most notably, in that only a small (∼20%) reduction is observed when wet NH_3_ is used and that the MOF remains stable under these conditions as confirmed by PXRD (Figure S17).

## Conclusions

The dual-functionalized MFM-303(Al) material has shown reversible adsorption of NH_3_ with an exceptional packing density of 0.801 cm^–3^ within the pores at 293 K, comparable to that of the solid NH_3_. The strong interaction of the framework with NH_3_ has been revealed by *in situ* synchrotron PXRD and INS/DFT studies. MFM-303(Al) illustrates the importance of the free carboxylic and available hydroxyl groups in binding substrates, and demonstrates the potential of integrating efficient storage and packing with dynamic capture within functional MOF materials.

## Supporting Information Available

Experimental methods and characterization, additional details of structure analysis, gas isotherms and analysis, and breakthrough experiments (PDF)

## Accession Codes

CCDC 1491199 and 2061871 contain the supplementary crystallographic data for this paper. These data can be obtained free of charge via www.ccdc.cam.ac.uk/data_request/cif, or by emailing data_request@ccdc.cam.ac.uk, or by contacting The Cambridge Crystallographic Data Centre, 12 Union Road, Cambridge CB2 1EZ, UK; fax: +44 1223 336033.
