# Supplementary material for: Exceptional Packing Density of Ammonia in a Dual-Functionalized Metal–Organic Framework
Source: J Am Chem Soc. 2021 Apr 22;143(17):6586–92. doi: 10.1021/jacs.1c01749 (PMC8154541; doi:10.1021/jacs.1c01749)
Supplement: Supplementary file 1 — ja1c01749_si_001.pdf [file ja1c01749_si_001.pdf]

Supporting Information

Exceptional packing density of ammonia in a dual-functionalised metal-organic framework

Christopher Marsh[1], Xue Han[1], Jiangnan Li[1], Zhenzhong Lu[1], Stephen P. Argent[2], Ivan da Silva[3], Yongqiang Cheng[4], Luke L. Daemen[4], Anibal J. Ramirez-Cuesta[4], Stephen P. Thompson[5], Alexander J. Blake[2], Sihai Yang[1]* and Martin Schröder[1]*

1. Department of Chemistry, University of Manchester, Manchester, M13 9PL, UK
2. School of Chemistry, University of Nottingham, Nottingham, NG7 2RD, UK
3. ISIS Neutron and Muon Source, Rutherford Appleton Laboratory, Oxford, OX11 0QX, UK
4. Neutron Scattering Division, Neutron Sciences Directorate, Oak Ridge National Laboratory, Oak Ridge, TN 37831, USA
5. Diamond Light Source, Harwell Science Campus, Oxfordshire, OX11 0DE, UK.

1. Experimental methods and procedures S3

2. Crystallographic data for MFM-303(Al) S4

3. Solvent and pH stability tests S8

4. N_2 adsorption isotherm S9

5. CO_2 isotherm and DFT/Monte-Carlo fitting for surface area and pore volume determination S10

6. Additional NH_3 isotherms S11

7. Water sorption isotherm S11

8. Thermogravimetric analysis S12

9. Differential scanning calorimetry S14

10. FTIR-breakthrough experiments S15

11. Density function theoretical (DFT) calculations S16

12. Additional views of the structure of MFM-303(Al) S17

13. Stability of MFM-303(Al) after breakthrough experiments S18

14. Formation of defects in MFM-303(Al) S19

15. Comparison of MFM-303(Al) to other materials S21

16. Supplementary references S21

1. Experimental methods and procedures

NH_3-ABR-TPD experiments. The sample was placed into a packed bed reactor of the lab-developed TPD equipment equipped with a Bruker Matrix MG5 FTIR spectrometer (resolution = 0.5 cm^{-1}) for gas analysis and was first activated at 120 °C under He at 100 mL min^{-1} (99.999%, BOC gas Ltd.) for 20 h in order to remove adsorbed guest molecules. After the activation, the temperature of the sample was decreased to 25 °C and the temperature maintained until a stable IR baseline signal was achieved. Then a gas mixture of 5000 ppm NH_3 diluted in He was introduced to establish the saturated NH_3 signal (i.e. the breakthrough of NH_3 adsorption). After that, the gas was switched to pure He (at 100 mL min^{-1}) to purge the physically adsorbed NH_3 from the sample until obtaining a stable NH_3 signal. The temperature of the reactor was then increased at a rate of 5 °C min^{-1} whilst maintaining a flow of pure He to measure the NH_3 desorption.

2. Crystallographic data for MFM-303(Al)

Supplementary Table S1. Summary of crystallographic data for as-synthesised MFM-303(Al)

Crystal data	
Chemical formula	$C_{16}H_8AlO_9 \cdot 2.75(H_2O)$
M_r	421.76
Crystal system, space group	Monoclinic, *C*2/*c*
Temperature (K)	120
a, *b*, *c* (Å)	13.705(3), 20.837(2), 13.2871(12)
β (°)	92.544(13)
V ($Å^3$)	3790.7(11)
Z	8
Radiation type	synchrotron, λ = 0.6889(2) Å
μ (mm^{-1})	0.16
Crystal size (mm)	0.05 × 0.05 × 0.02
Data collection	
Diffractometer	Kappa Rigaku Saturn 724+
Absorption correction	Multi-scan. *CrysAlis PRO*, Agilent Technologies, Version 1.171.37.35 (release 13-08-2014 CrysAlis171 .NET) (compiled Aug 13 2014,18:06:01) Empirical absorption correction using spherical harmonics, implemented in SCALE3 ABSPACK scaling algorithm.
T_{min}, T_{max}	0.741, 1.000
No. of measured, independent and observed [$I > 2\sigma(I)$] reflections	16953, 2310, 1655
R_{int}	0.143
θ_{max} (°)	21.3
$(\sin \theta/\lambda)_{max}$ ($Å^{-1}$)	0.526
Refinement	
$R[F^2 > 2\sigma(F^2)]$, $wR(F^2)$, S	0.148, 0.410, 1.64
No. of reflections, no. of parameters, no. of restraints	2310, 281, 355
H-atom treatment	H atoms treated by a mixture of independent and constrained refinement
Q_{max}, Q_{min} (e $Å^{-3}$)	1.26, -0.67

Computer programs: *CrysAlis PRO*, Agilent Technologies, Version 1.171.37.35 (release 13-08-2014 CrysAlis171.NET) (compiled Aug 13 2014, 18:06:01), *SHELXS*,[1] *SHELXL*,[2] Olex2[3]

Details of refinement of the single crystal structure of MFM-303(Al).

The X-ray diffraction of the crystal had a resolution limit of around 0.95 Å, and data beyond this resolution were omitted from the refinement using a SHEL command. As a consequence the structure has a low data to parameter ratio, and so where possible restraints were applied to the geometries and atomic displacement parameters of the atoms to aid refinement. The possibility of further unmodelled disorder in the ligand position is suggested by the shape of the displacement ellipsoids and large U3/U1 ratio for the average U(i,j) tensor (4.8). However, more complex disorder models did not yield a plausible structure or better fit with the data. Rigid bond restraints have been applied to the anisotropic displacement parameters of all atoms in the structure (RIGU).

The carboxylic acid group C61-O63 is disordered over two orientations whose occupancies have been refined and constrained to sum to unity, resulting in values of 0.61(5) and 0.39(5). The position and anisotropic displacement parameters of the carbon atom of each disorder component have been constrained to be identical

(EAXY, EADP). The 1,2 and 1,3 bond distances of the two components have been restrained to be similar (SAME) and each component has been constrained to have planar geometry coincident with phenyl carbon atom C25 (FLAT). The anisotropic displacement parameters of the disordered oxygen atoms have been restrained to be similar and isotropic (SIMU, ISOR).

The isotropic displacement parameters of water residues O1W, O2W and O3W were fixed to have values of 0.08 whilst their occupancies were allowed to refine resulting in values of 0.94(1), 0.48(1) and 0.39(1) respectively. The water residues have been included in the unit cell contents at full occupancy. Hydrogen atoms on the phenyl rings were geometrically placed and refined with a riding model. Electron density peaks in suitable positions for hydrogen atoms were observed close to the hydroxyl-oxygen atom O1H, carboxylic acid hydroxyl-oxygen atom O42 and the water residue O2W. The positions of these hydrogen atoms were allowed to refine under the influence of geometric restraints. Further electron density peaks were observed close to water residues O1W and O3W; however, they could not be developed into sensible models for hydrogen atoms. These potential hydrogen atoms were not included in the model, but were included in the unit cell contents at full occupancy.

The hydroxyl hydrogen position at O1H is disordered over two positions with each refined to have half occupancy. The O-H bond lengths were restrained to suitable target values (DFIX) whilst all the H...Al 1,3 distances were restrained to be the same (SADI). Their isotropic displacement parameters were fixed at a value of 0.1. The positions of both atoms were weakly restrained to donate hydrogen bonds to water residue O2W. The 1,2 and 1,3 distances of the hydrogen atoms of water residue O2W were restrained to have suitable values (DFIX). Further weaker restraints were applied to their positions to make them donate hydrogen bonds to adjacent water residue O3W and carboxylic acid oxygen O63A. Their isotropic displacement parameters were fixed at a value of 0.12. An electron density peak was observed almost equidistant from, and directly between carboxylic acid oxygen atoms O42 and O62A. The electron density was modelled as a full occupancy hydrogen atom and its position allowed to refine freely. The isotropic displacement parameter of the hydrogen atom was fixed at 1.5 x Ueq of oxygen atom O42. Further disordered solvent molecules could not be sensibly modelled, so the structure was treated with PLATON SQUEEZE. A total of 32 electrons were accounted from the P1 cell in this, equating to a quarter of a water molecule per asymmetric unit, which have been included in the unit cell contents and calculation of derived parameters.

Supplementary Table S2. Summary of crystallographic data of MFM-303(Al)·$4.36NH_3$.

Chemical formula	$C_{16}H_8AlO_9 \cdot 4.36NH_3$
M_r	423.33
Crystal system, space group	Monoclinic, *C*2/*c*
Temperature (K)	273
a, *b*, *c* (Å)	15.52974(17), 19.62387(17), 13.14259(12)
β (°)	92.3435(8)
V (Å^3)	4001.90(7)
Z	8
Radiation type	synchrotron, λ = 0.825774(2) Å
R_{wp}	7.54%
R_{exp}	1.63%
R_p	5.54%
GoF	4.62

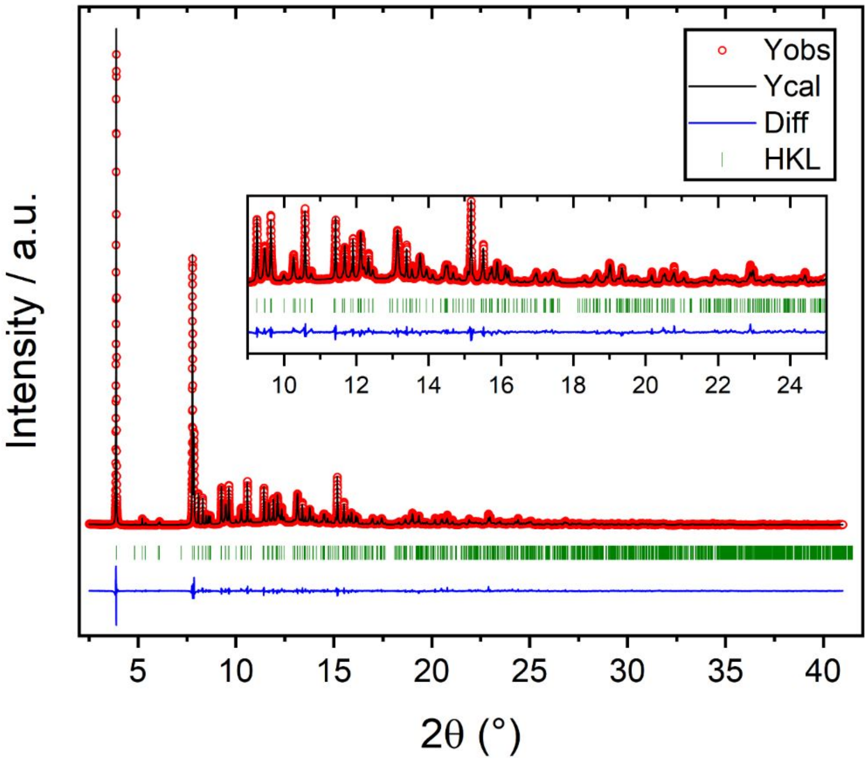

Supplementary Figure S1. Rietveld refinement graph for NH_3@MFM-303(Al) (λ = 0.825774 Å).

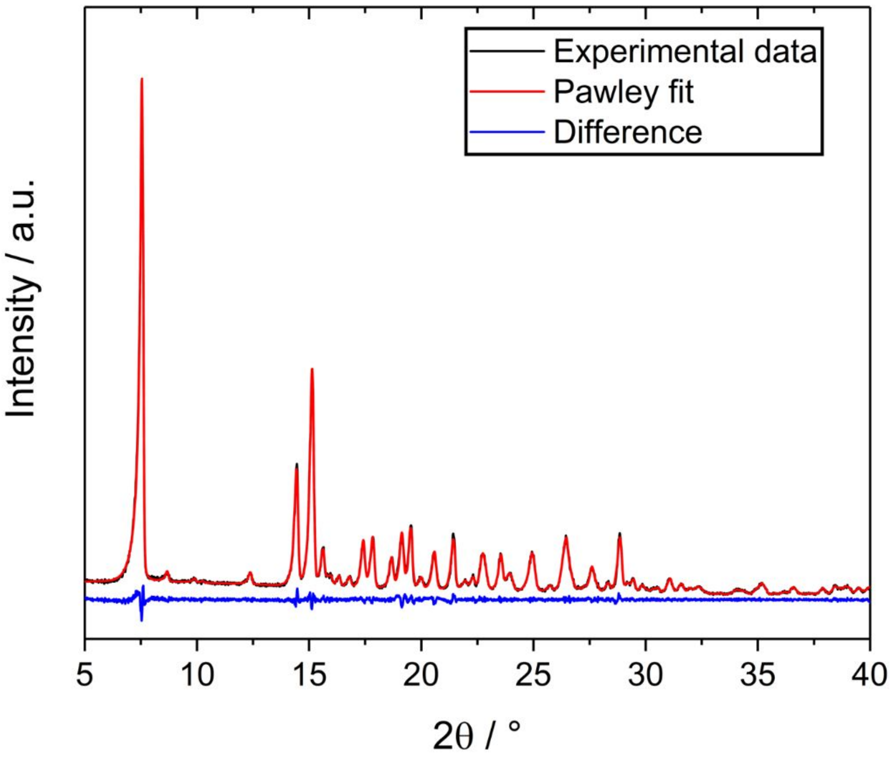

Supplementary Figure S2. Pawley fitting of bulk sample of MFM-303(Al) confirming the phase purity of the material (λ = 1.5406 Å, R_p=4.13%, R_{wp} = 5.31%, goodness-of-fit = 1.37)

3. Solvent and pH stability tests

a

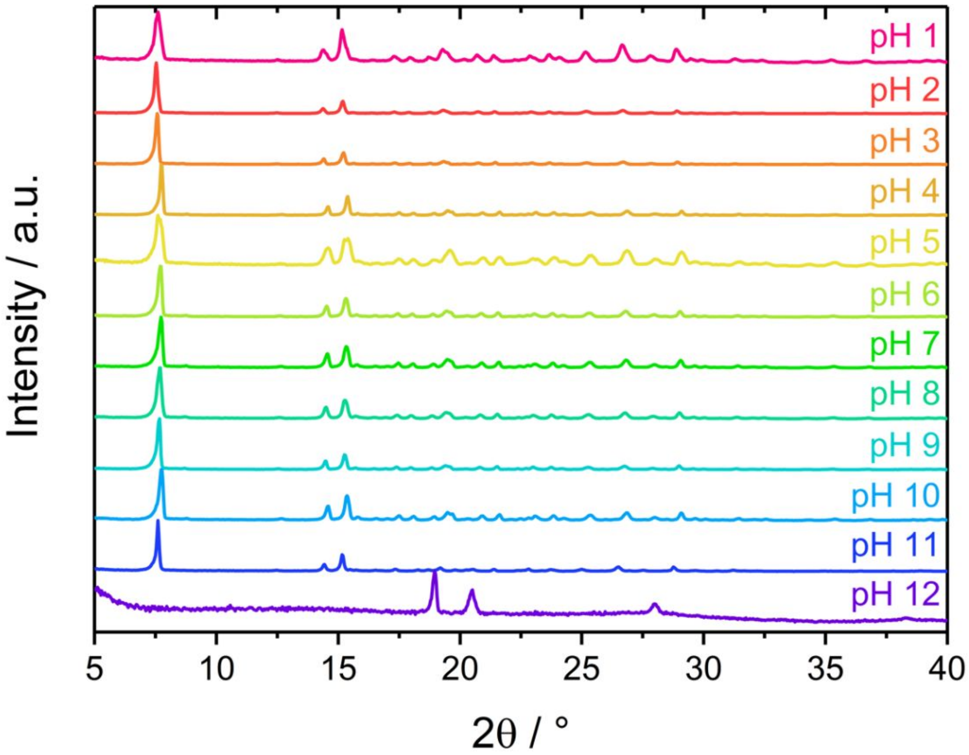

b

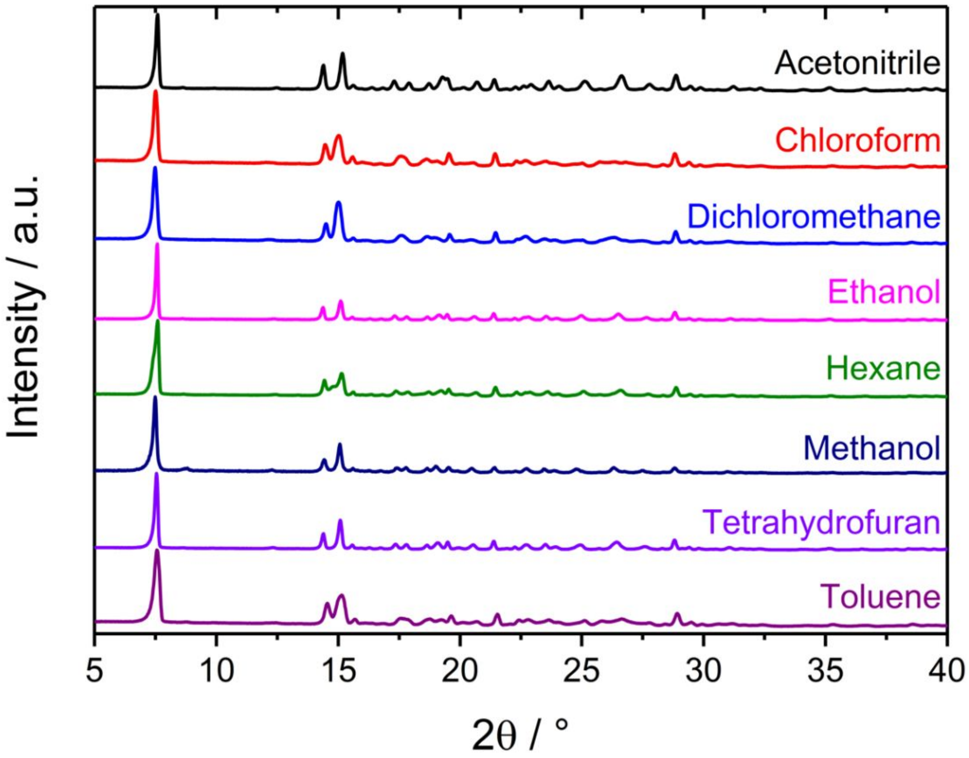

Supplementary Figure S3. Stability of MFM-303(Al) under different organic and aqueous solvents measured by PXRD. **a**, PXRD patterns of MFM-303(Al) after immersion in aqueous solutions of different pH for 16 h. **b**, PXRD patterns of MFM-303(Al) after immersion in different solvents for 16 h.

4. N_2 adsorption isotherm

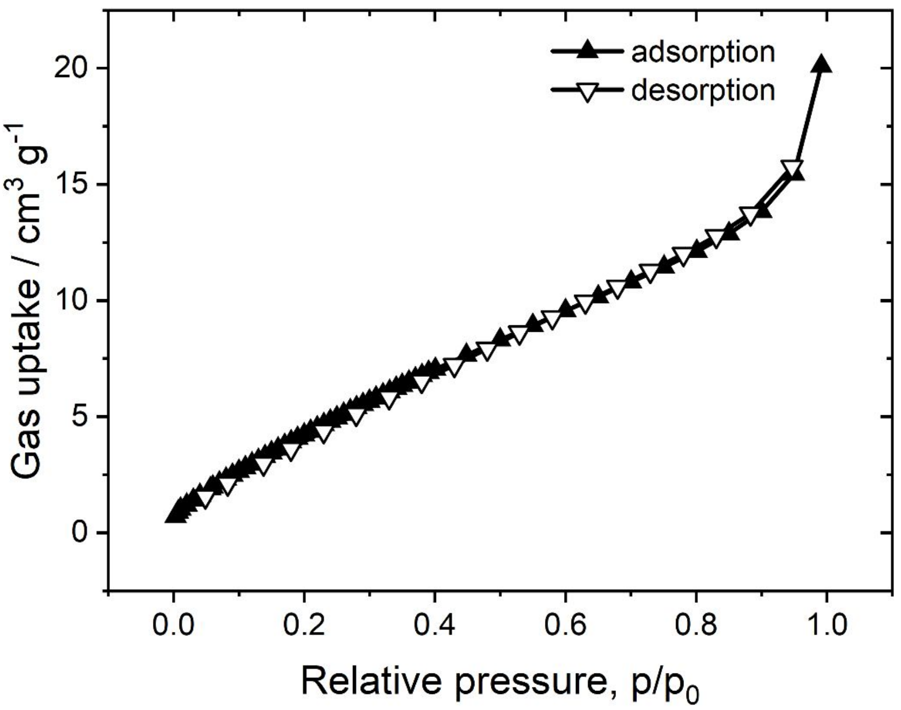

Supplementary Figure S4. Adsorption isotherm of N_2 in MFM-303(Al) at 77 K demonstrating poor uptake most likely due to activation diffusion effects of N_2 molecules through the narrow pores of MFM-303(Al).

5. CO_2 isotherms and DFT/Monte-Carlo fitting for surface area and pore volume determination

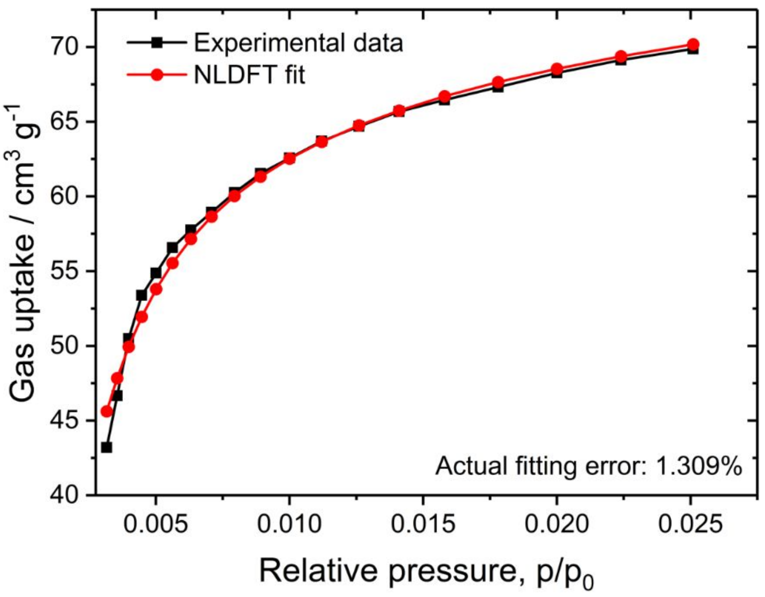

Supplementary Figure S5. DFT/Monte-Carlo fitting for the adsorption isotherm for CO_2 in MFM-303(Al) at 273 K; this gave a surface area of 723 m^2 g^{-1} and pore volume of 0.150 cm^3 g^{-1} based upon the CO_2 isotherm.[4]

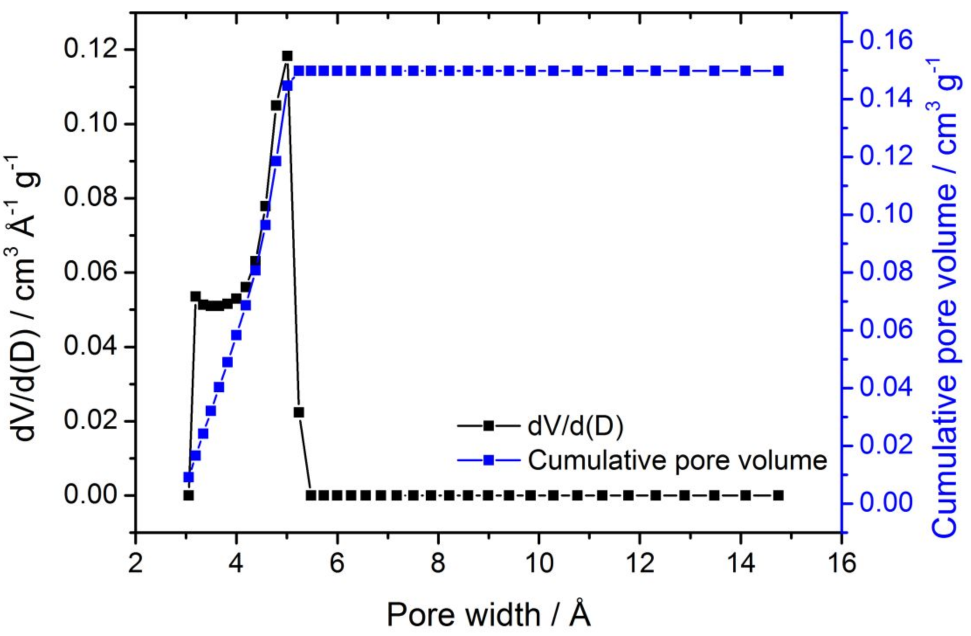

Supplementary Figure S6. DFT/Monte-Carlo pore volume distribution plot for MFM-303(Al) to give a pore diameter centred at ~5.0 Å.

6. Additional NH_3 isotherms

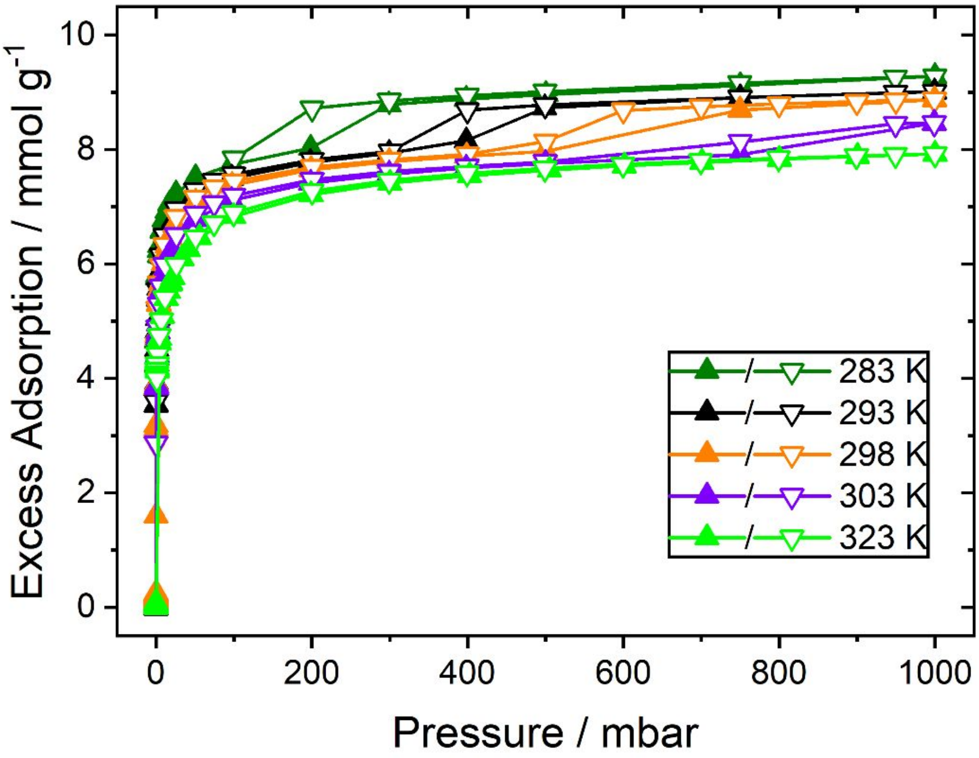

Supplementary Figure S7. Adsorption isotherms for NH_3 in MFM-303(Al).

7. Water sorption isotherm

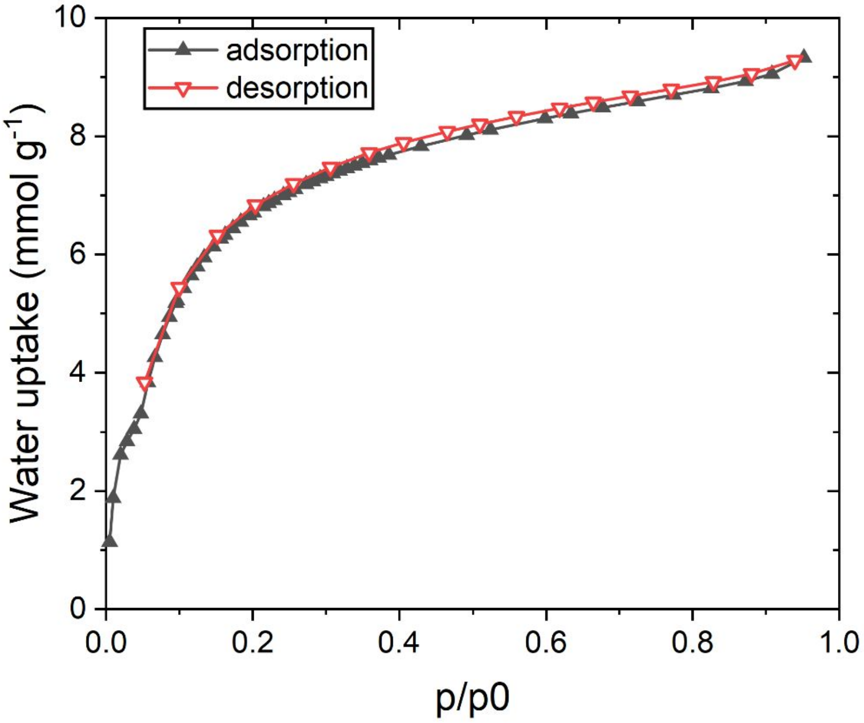

Supplementary Figure S8. Adsorption isotherm for water in MFM-303(Al) at 283 K.

8. Thermogravimetric analysis

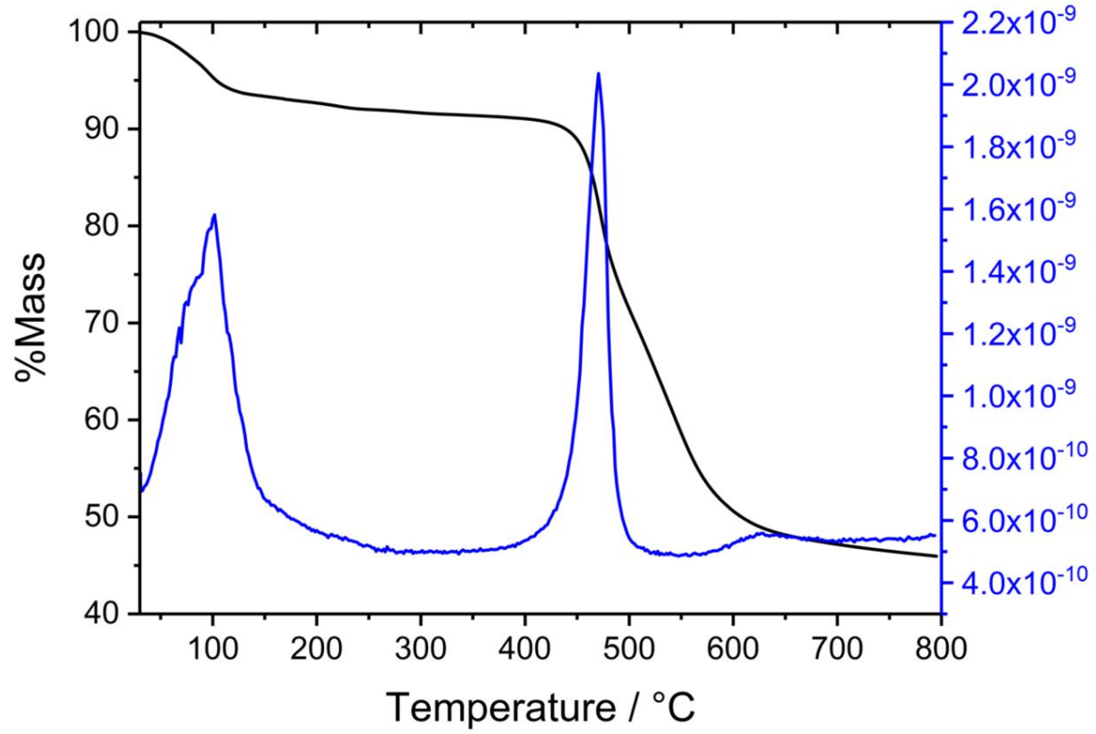

Supplementary Figure S9. Thermogravimetric analysis of as-synthesised MFM-303(Al) under a flow of N_2 at a heating rate of 10 °C min^{-1} with mass spectrometric data for the peak at m/z = 18 (corresponding to water).

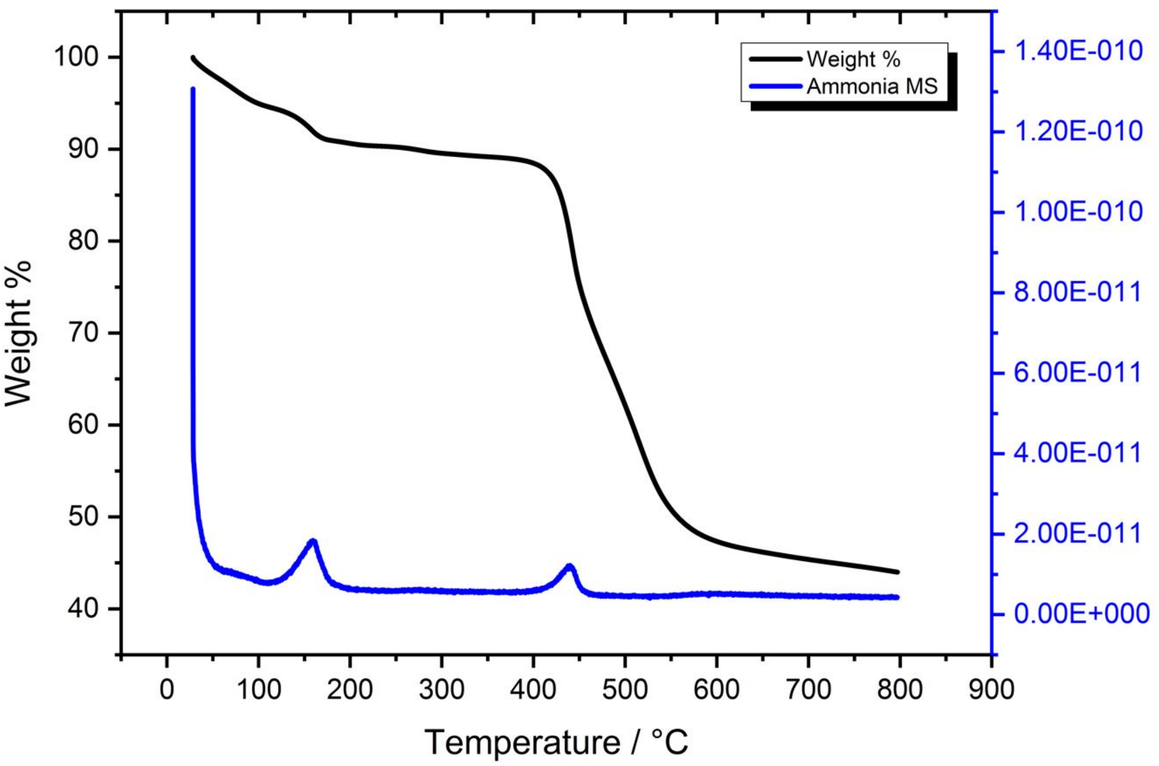

Supplementary Figure S10. TGA-MS data for MFM-303(Al) pre-loaded with NH_3. The sample was activated at 150 °C under vacuum and then exposed to NH_3 at 1 bar and excess gas removed. The sample was then transferred to the TGA-MS. A peak at 113 °C and ending at 190 °C corresponds to a weight loss of 94.55-90.80 = 3.75, equating to 2.42 mmol g^{-1}.

9. Differential scanning calorimetry

Differential scanning calorimetry was performed on a Thermal Analysis SDT650 analyser, and was used to determine the enthalpy of adsorption. The sample was heated under a flow of N_2 (50 mL min^{-1}) at 200 °C for 2 h to remove adsorbed solvent molecules. After cooling to 30 °C, NH_3 (0.5% diluted in He) was introduced at a flow rate of 10 mL min^{-1} and the sample allowed to equilibrate. The heat of adsorption was calculated by integrating the area under the normalised heat flow curve.

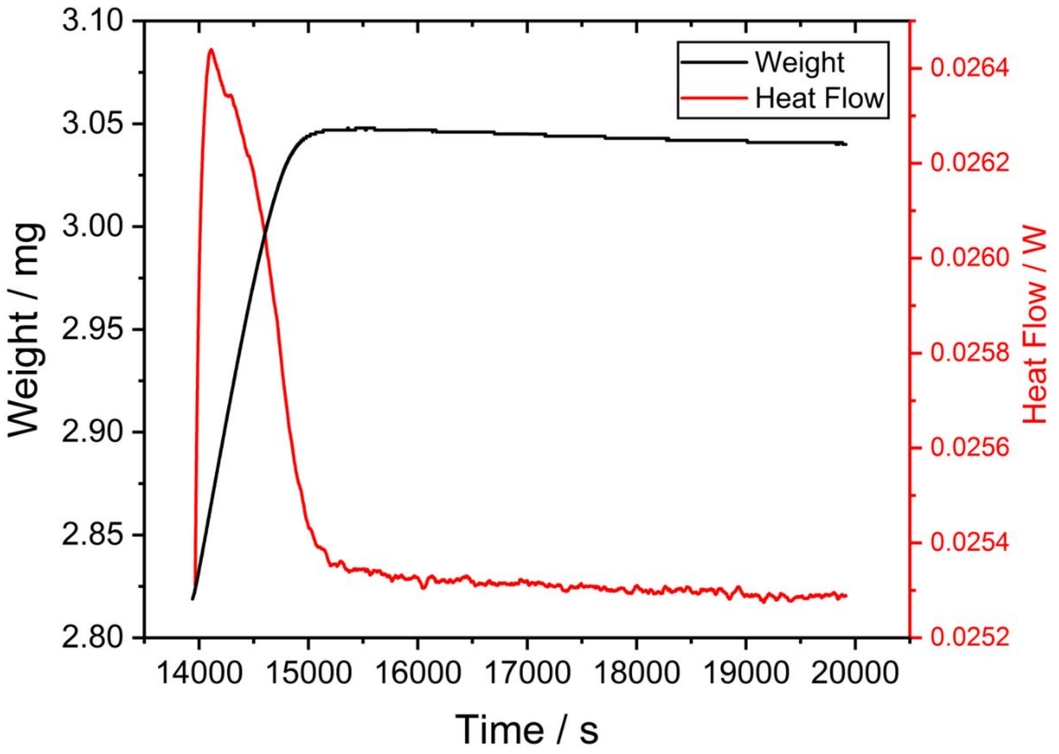

Supplementary Figure S11. DSC curve for activated MFM-303(Al) under NH_3 (0.5% diluted in He) at a flow rate of 10 mL min^{-1}. The enthalpy of adsorption is calculated to be 61.5 kJ mol^{-1}.

10. FTIR-breakthrough experiments

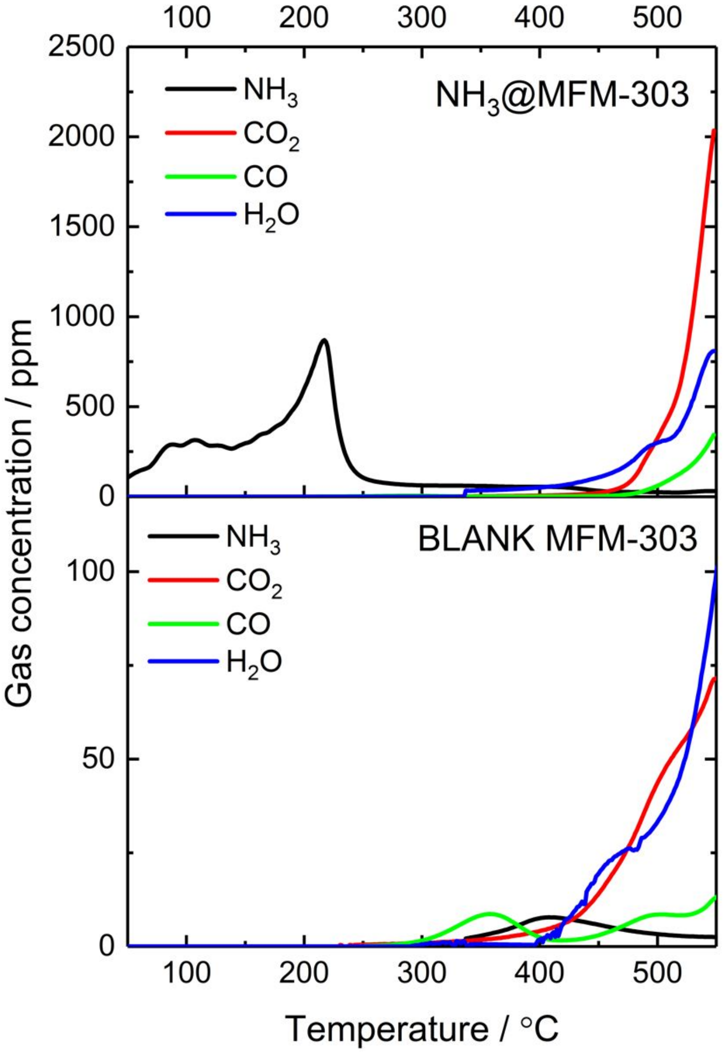

Supplementary Figure S12. Temperature-programmed desorption (TPD) experiments performed on NH_3 in MFM-303(Al) using FTIR to detect the gases eluting from the column.

11. Density function theoretical (DFT) calculations

Modelling by Density Functional Theory (DFT) of the bare and NH_3/CO_2-loaded MOFs was performed using the Vienna Ab initio Simulation Package (VASP).[5] The calculation used the Projector Augmented Wave (PAW) method[6,7] to describe the effects of core electrons, and Perdew-Burke-Ernzerhof (PBE)[8] implementation of the Generalized Gradient Approximation (GGA) for the exchange-correlation functional. Energy cutoff was 800 eV for the plane-wave basis of the valence electrons. The lattice parameters and atomic coordinates determined by X-ray single crystal diffraction in this work were used as the initial structure. Due to the large unit cell (~300 atoms), all electronic structure and phonon calculations were performed on the Γ point only. The total energy tolerance for electronic energy minimization was 10^{-8} eV, and for structure optimization it was 10^{-7} eV. The maximum interatomic force after relaxation was below 0.001 eV/Å. The optB86b-vdW functional[9] for dispersion corrections was applied. The vibrational eigen-frequencies and modes were then calculated by solving the force constants and dynamical matrix using Phonopy.[10] The OClimax software[11,12] was used to convert the DFT-calculated phonon results to the simulated INS spectra. Simulation of the INS spectra for NH_3-loaded MFM-303(Al) was conducted with NH_3 presenting at site II due to its strong interaction with the –OH group of the framework. The simulation of NH_3 at site I breaks the symmetry of the model and unfortunately makes the calculation impractical.

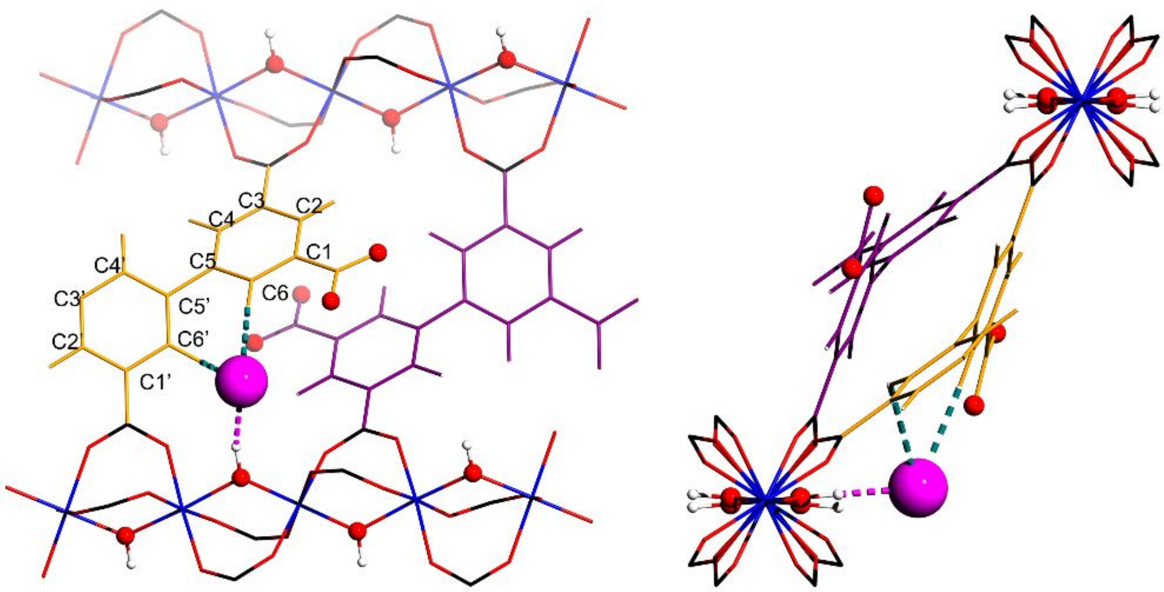

Supplementary Figure S13. View of NH_3 site II derived from the model used to simulate INS spectra and its proximity to carbon atoms on the aromatic ring and to the μ-OH moiety.

12. Additional views of the structure of MFM-303(Al)

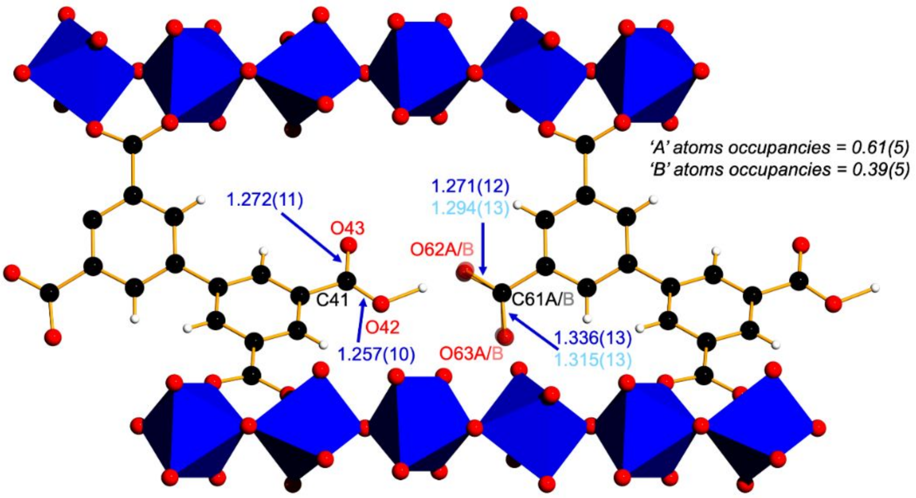

Supplementary Figure S14. View of the structure of MFM-303(Al) showing the C-O and C=O bond lengths in adjacent ligand units and disordered atoms O62, O63 and C61.

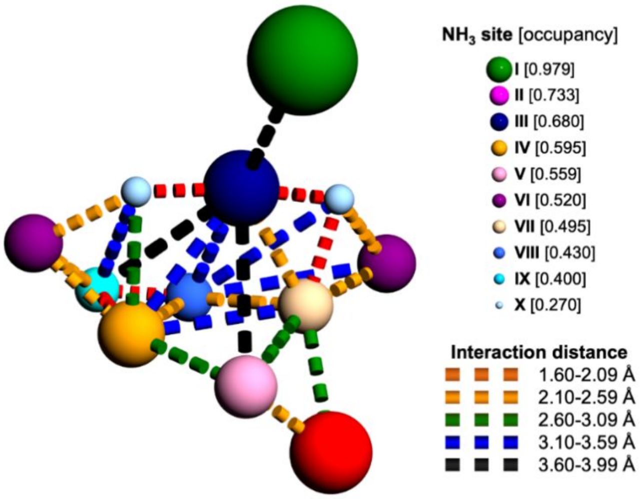

Supplementary Figure S15. View of NH_3 sites in NH_3@MFM-303(Al) showing intermolecular distances (N atom size and colour corresponds to site number). Colour of bonds denote the intermolecular distance.

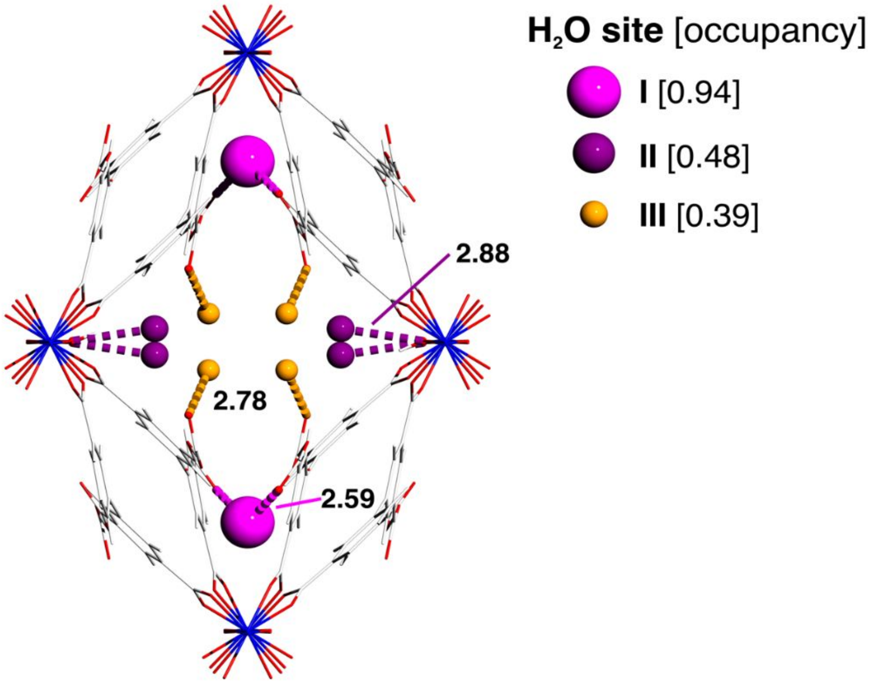

Supplementary Figure S16. View of the as-synthesised MFM-303(Al) containing water (O atoms shown only for clarity), highlighting O···O intermolecular distances.

13. Stability of MFM-303(Al) after breakthrough experiments

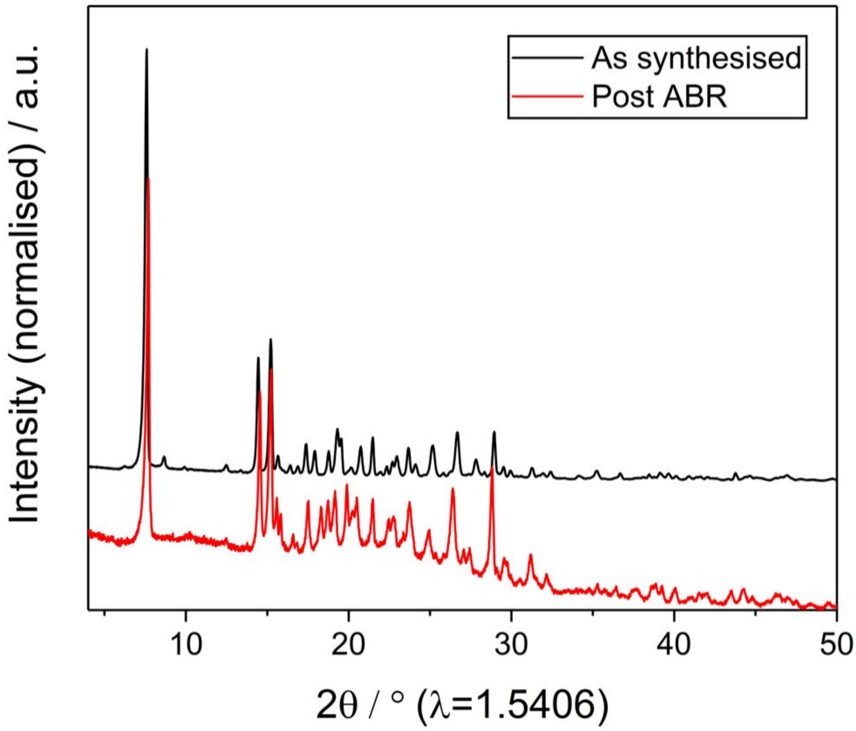

Supplementary Figure S17. PXRD patterns for as-synthesised MFM-303(Al) and after breakthrough with dry and then wet NH_3, demonstrating the stability of this material.

14. Formation of defects in MFM-303(Al)

One possible explanation for the high density of NH_3 in MFM-303(Al) is the presence of defects within the material. The determination of defects typically relies on a combination of methods to characterise. For MFM-303(Al), PXRD data provided little evidence; the bulk material was refined using a Pawley fit and no unexpected peaks or features in the baseline were observed (Figure S2). To investigate the possible presence of defects, 100 mg MFM-303(Al) was boiled at 120 °C in DMF overnight then boiled in MeOH at 70 °C for 1 day. A small sample (~5 mg) was completely dissolved using a digestion solution (20 μL trifluoroacetic acid in 600 μL DMSO-d6) and ^{1}H NMR spectrum obtained with a Bruker Avance 400 MHz spectrometer. As shown in Figure S18, the ligand peaks are present as is some MeOH. No other peaks are observed suggesting there is little evidence for other species, such as formate from DMF, binding to potential defect sites such as those missing cluster units.

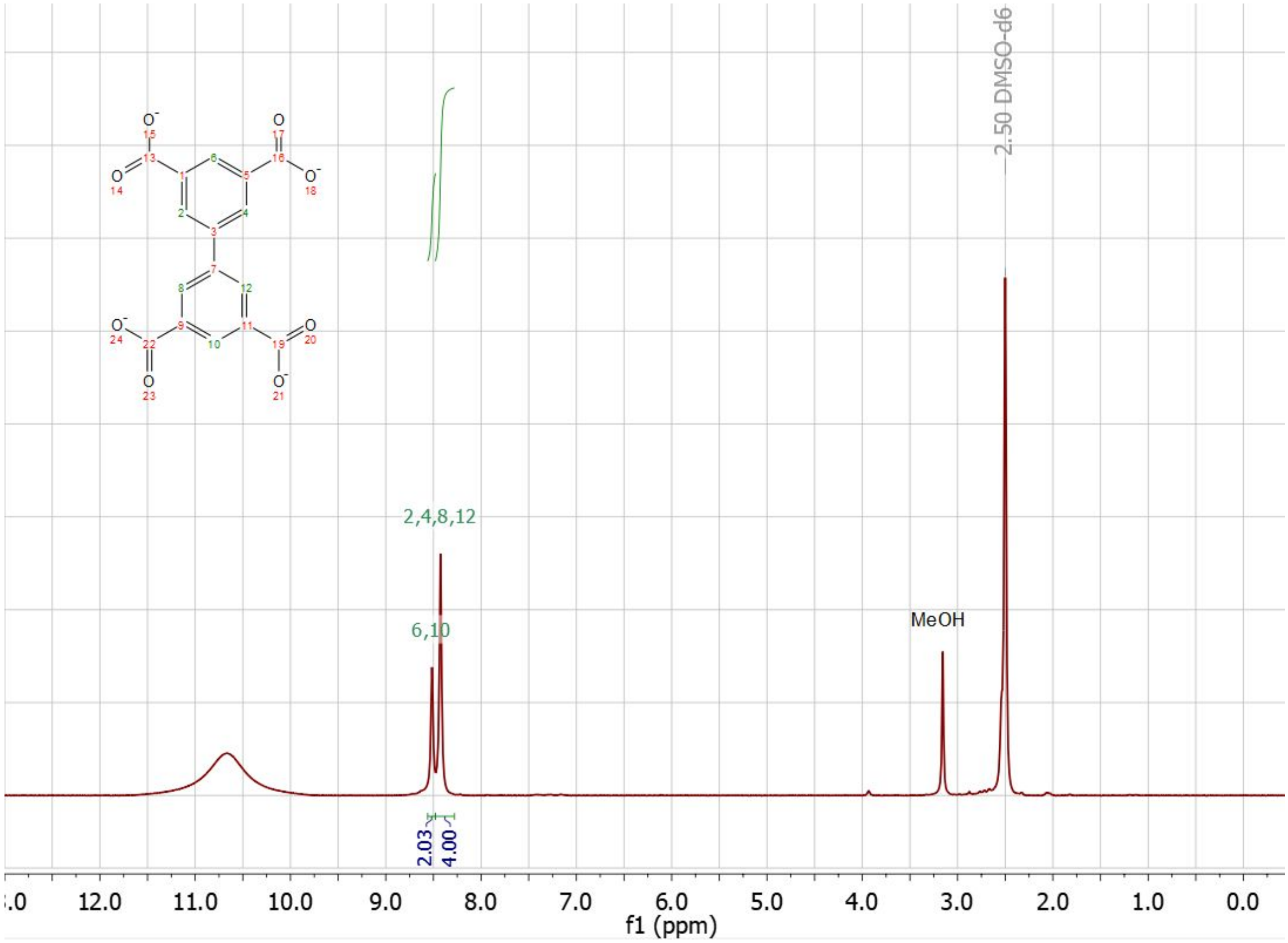

Supplementary Figure S18. ^{1}H NMR spectrum of digested MFM-303(Al) sample after methanol exchange.

Thermogravimetric analysis in air was also used to probe the possibility of defects (Figure S19). Assuming complete thermal decomposition to Al_2O_2, a 1:0.50 ratio of MOF:Al_2O_3 would be expected. The actual ratio was found to be 1:0.52, which could suggest the presence of trace amount of missing linker.

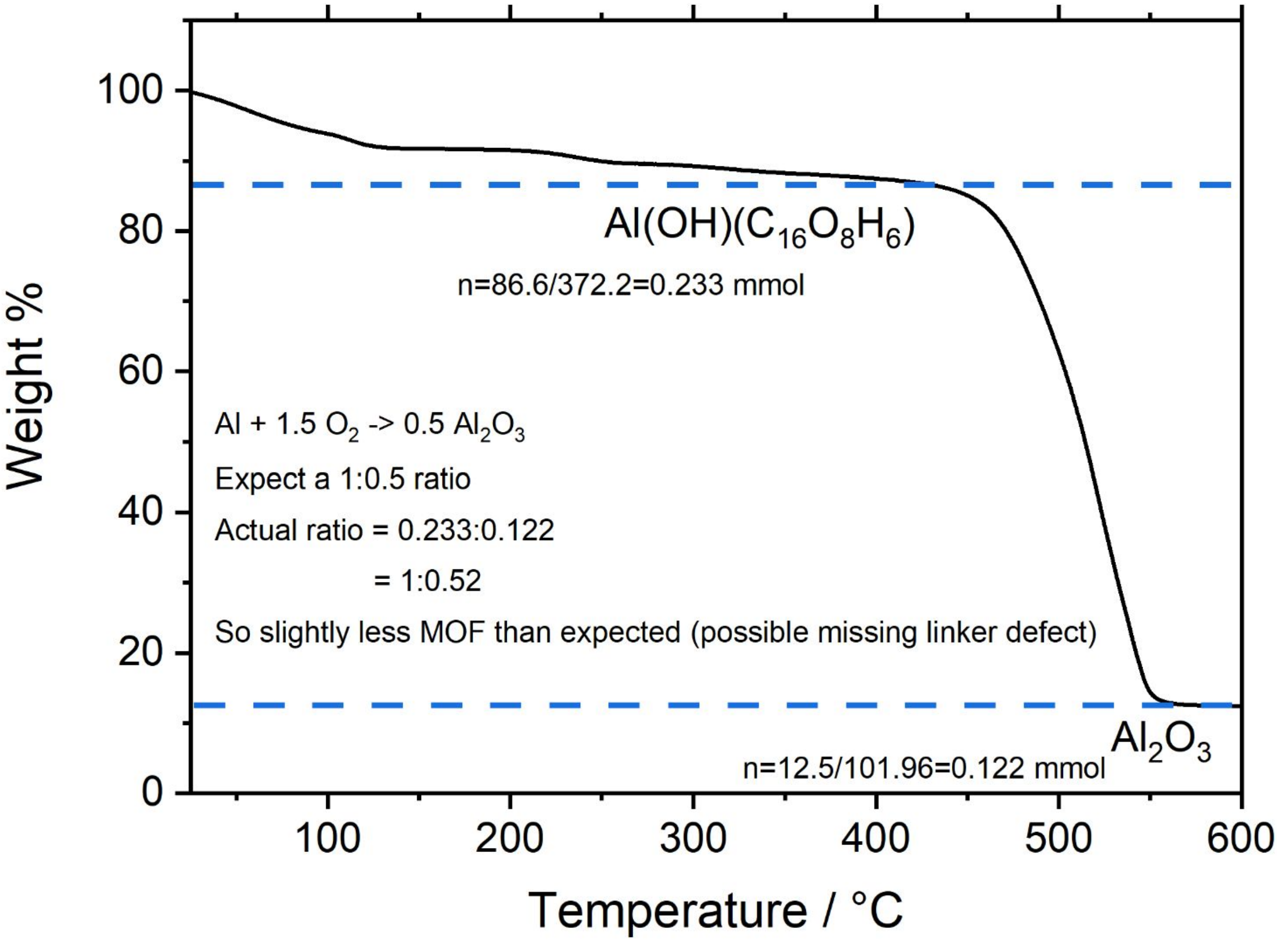

Supplementary Figure S19. TGA of MFM-303(Al) under a flow of air, suggesting that defects may be present but only at low concentration.

15. Comparison of MFM-303(Al) to other materials

Supplementary Table S3. Dynamic breakthrough capacity of porous materials compared with MFM-303(Al)

Material	NH_3 concentration (ppm)	Dynamic NH_3 capacity (mmol g^{-1})		Reduction in adsorption capacity	Stability after NH_3 sorption	Ref
		Dry conditions	Wet conditions			
MFM-303(Al)	833	2.9	2.4	17%	Stable	This work
MFM-300(V^{III})	1,000	1.9	(not measured)	-	Stable	13
Mg_2(dobpdc)	1,000	8.37	6.14	27%	Stable	14
Co_2Cl_2BTDD	1,000	4.78	3.38	29%	Unstable	15
Co_2Cl_2BBTA	1,000	8.56	4.36	49%	Unstable at 1 bar, partially stable at 1 mbar	15
Mg-MOF-74	1,440	7.60	1.70	78%	Not reported	16
UiO-66-OH	2,880	5.69	2.77	51%	Not reported	17
UiO-66-$(COOH)_2$	1,431	2.83	1.83	35%	Not reported	17
Zeolite 13X	1,440	2.86	0.62	78%	Not reported	16

*As determined by PXRD

16. Supplementary references

(1) Sheldrick, G. M. A Short History of SHELX. *Acta Crystallogr. Sect. A Found. Crystallogr.* **2008**, *64* (1), 112–122.

(2) Sheldrick, G. M. Crystal Structure Refinement with SHELXL. *Acta Crystallogr. Sect. C Struct. Chem.* **2015**, *71* (Md), 3–8.

(3) Dolomanov, O. V.; Bourhis, L. J.; Gildea, R. J.; Howard, J. A. K.; Puschmann, H. OLEX2: A Complete Structure Solution, Refinement and Analysis Program. *J. Appl. Crystallogr.* **2009**, *42* (2), 339–341.

(4) Yang, S.; Sun, J.; Ramirez-Cuesta, A. J.; Callear, S. K.; David, W. I. F.; Anderson, D. P.; Newby, R.; Blake, A. J.; Parker, J. E.; Tang, C. C.; Schröder, M. Selectivity and Direct Visualization of Carbon Dioxide and Sulfur Dioxide in a Decorated Porous Host. *Nat. Chem.* **2012**, *4* (11), 887–894.

(5) Kresse, G.; Furthmüller, J. Efficient Iterative Schemes for Ab Initio Total-Energy Calculations Using a Plane-Wave Basis Set. *Phys. Rev. B* **1996**, *54* (16), 11169–11186.

(6) Blöchl, P. E. Projector Augmented-Wave Method. *Phys. Rev. B* **1994**, *50* (24), 17953–17979.

(7) Kresse, G.; Joubert, D. From Ultrasoft Pseudopotentials to the Projector Augmented-Wave Method. *Phys. Rev. B* **1999**, *59* (3), 1758–1775.

(8) Perdew, J. P.; Burke, K.; Ernzerhof, M. Generalized Gradient Approximation Made Simple. *Phys. Rev. Lett.* **1996**, *77* (18), 3865–3868.

(9) Klimeš, J.; Bowler, D. R.; Michaelides, A. Chemical Accuracy for the van Der Waals Density Functional. *J. Phys. Condens. Matter* **2010**, *22* (2), 022201.

(10) Togo, A.; Tanaka, I. First Principles Phonon Calculations in Materials Science. *Scr. Mater.* **2015**, *108*, 1–5.

(11) Cheng, Y. Q.; Daemen, L. L.; Kolesnikov, A. I.; Ramirez-Cuesta, A. J. Simulation of Inelastic Neutron Scattering Spectra Using OCLIMAX. *J. Chem. Theory Comput.* **2019**, *15* (3), 1974–1982.

(12) Cheng, Y.; Daemen, L. L.; Kolesnikov, A. I.; Ramirez-Cuesta, A. J. OCLIMAX https://sites.google.com/site/ornliceman/download.

(13) Kim, D. W.; Kang, D. W.; Kang, M.; Lee, J.; Choe, J. H.; Chae, Y. S.; Choi, D. S.; Yun, H.; Hong, C. S. High Ammonia Uptake of a Metal–Organic Framework Adsorbent in a Wide Pressure Range. *Angew. Chemie Int. Ed.* **2020**, *59* (50), 22531–22536.

(14) Han, X.; Lu, W.; Chen, Y.; da Silva, I.; Li, J.; Lin, L.; Li, W.; Sheveleva, A. M.; Godfrey, H. G. W.; Lu, Z.; Tuna, F.; McInnes, E. J. L.; Cheng, Y.; Daemen, L. L.; MPherson, L. J. M.; Teat, S. J.; Frogley, M. D.; Rudić, S.; Manuel, P.; Ramirez-Cuesta, A. J.; Yang, S.; Schröder, M. High Ammonia Adsorption in MFM-300 Materials: Dynamics and Charge Transfer in Host–Guest Binding. *J. Am. Chem. Soc.* **2021**, *143* (8), 3153–3161.

(15) Rieth, A. J.; Dincă, M. Controlled Gas Uptake in Metal–Organic Frameworks with Record Ammonia Sorption. *J. Am. Chem. Soc.* **2018**, *140* (9), 3461–3466.

(16) Grant Glover, T.; Peterson, G. W.; Schindler, B. J.; Britt, D.; Yaghi, O. MOF-74 Building Unit Has a Direct Impact on Toxic Gas Adsorption. *Chem. Eng. Sci.* **2011**, *66* (2), 163–170.

(17) Jasuja, H.; Peterson, G. W.; Decoste, J. B.; Browe, M. A.; Walton, K. S. Evaluation of MOFs for Air Purification and Air Quality Control Applications: Ammonia Removal from Air. *Chem. Eng. Sci.* **2015**, *124*, 118–124.
